# Disseminated Blastomycosis With Pulmonary, Cutaneous, and Splenic Involvement in a Construction Worker: A Case Report

**DOI:** 10.1002/ccr3.73710

**Published:** 2026-10-10

**Authors:** Toan N. Vu, Nilay Kumar

**Affiliations:** ^1^ University of Wisconsin School of Medicine and Public Health Madison Wisconsin USA; ^2^ Division of Hospital Medicine, Department of Medicine University of Wisconsin School of Medicine and Public Health Madison Wisconsin USA

**Keywords:** blastomycosis, case report, disseminated blastomycosis, fungal infection, occupational health

## Abstract

Blastomycosis is a systemic fungal infection that is caused by *Blastomyces dermatitidis* or *Blastomyces gilchristii*. Although the lungs are the primary site of infection, blastomycosis can disseminate to the skin, bones, genitourinary system, and, rarely, visceral organs such as the spleen. Occupational exposure to soil and decaying matter, particularly in construction workers, increases the risk of blastomycosis infection. A 40‐year‐old male presented with a progressively enlarging right facial lesion, which initially appeared as a small papule. The lesion had gradually increased in size and pain over several months and failed to respond to antibiotics. Notable symptoms include generalized fatigue, low mood, intermittent morning headaches, confusion, poor appetite with weight loss, intermittent night sweats, and joint pain. The diagnostic workup included imaging findings of an exophytic skin lesion arising from the right cheek, pulmonary micronodules and consolidation, and splenic changes. Serum *Blastomyces* antigen testing was positive, fungal culture of tissue from the facial lesion grew *Blastomyces dermatitidis*, and histopathologic examination demonstrated granulomatous inflammation with broad‐based budding yeast within the dermis, supporting the diagnosis of disseminated blastomycosis. Liposomal amphotericin B induction therapy was initiated, and the facial lesion improved. The patient was discharged home with a transition to oral itraconazole. This report presents a case of disseminated blastomycosis involving the lungs, skin, and spleen in an immunocompetent individual. High index of suspicion and recognition of cutaneous lesions in the context of occupational and environmental risk factors for blastomycosis enable early diagnosis and treatment.

## Key Clinical Message

Recognition of cutaneous manifestations in the setting of occupational and environmental risk factors is critical to early diagnosis and treatment of disseminated blastomycosis. Thorough occupational and exposure history, along with a high index of clinical suspicion, ensures timely diagnosis and prompt antifungal treatment, preventing life‐threatening complications.

**Abbreviations**
CNS: central nervous system
CT: computed tomography
mg: milligrams
mg/kg: milligrams per kilogram

## 1 Introduction

Blastomycosis is a systemic fungal infection caused by the dimorphic fungi *Blastomyces dermatitidis* or *Blastomyces gilchristii* [1]. It is endemic to specific parts of North America, such as regions surrounding the Ohio and Mississippi River valleys and Great Lakes [1]. A hallmark of blastomycosis is that it can be life‐threatening for both immunocompromised patients and immunocompetent patients [1, 2]. Although the lungs are the primary site of infection, blastomycosis can disseminate to the skin, bones, and genitourinary system, and less commonly to visceral organs such as the spleen [1]. Occupational and environmental exposures play a key role in transmission risk. Individuals exposed to soil and decaying matter, particularly those who do construction work or other soil‐disrupting activities, are at increased risk of exposure to *Blastomyces* spores [1, 3]. Blastomycosis is not transmitted person‐to‐person, underscoring the role of environmental exposure in disease acquisition [4]. Here, we present a case of disseminated blastomycosis extending beyond the lungs to involve the skin and spleen in a previously healthy individual.

## 2 Case Presentation

A 40‐year‐old male with no significant past medical history presented with a progressively enlarging right facial lesion, which initially appeared as a small papule (Figure 1). Over the course of several months, the lesion had gradually increased in size and pain, reaching a pain severity of 8 out of 10, and failed to respond to multiple courses of antibiotic therapies, including amoxicillin‐clavulanate, trimethoprim‐sulfamethoxazole, and ciprofloxacin. The patient's symptoms were notable for generalized fatigue, low mood, intermittent morning headaches, confusion, poor appetite with weight loss, intermittent night sweats, and joint pain. The patient denied any fevers, chills, changes in vision or taste, vomiting, diarrhea, constipation, or bloating. Prior to the onset of the lesion, the patient had a history of chest pain, dyspnea, and cough following a wisdom tooth extraction, all of which resolved before the lesion appeared. Occupational history was significant for construction work involving frequent exposure to soil and debris. The patient had no history of tobacco or alcohol use. The patient had recently moved from Mexico to Wisconsin and denied similar symptoms among family members or pets. Given the patient's occupational history, associated systemic symptoms, failure to respond to antibiotic therapy, and progressive nature of the facial lesion, further evaluation was pursued to assess for an atypical or disseminated infectious process.

**FIGURE 1:**
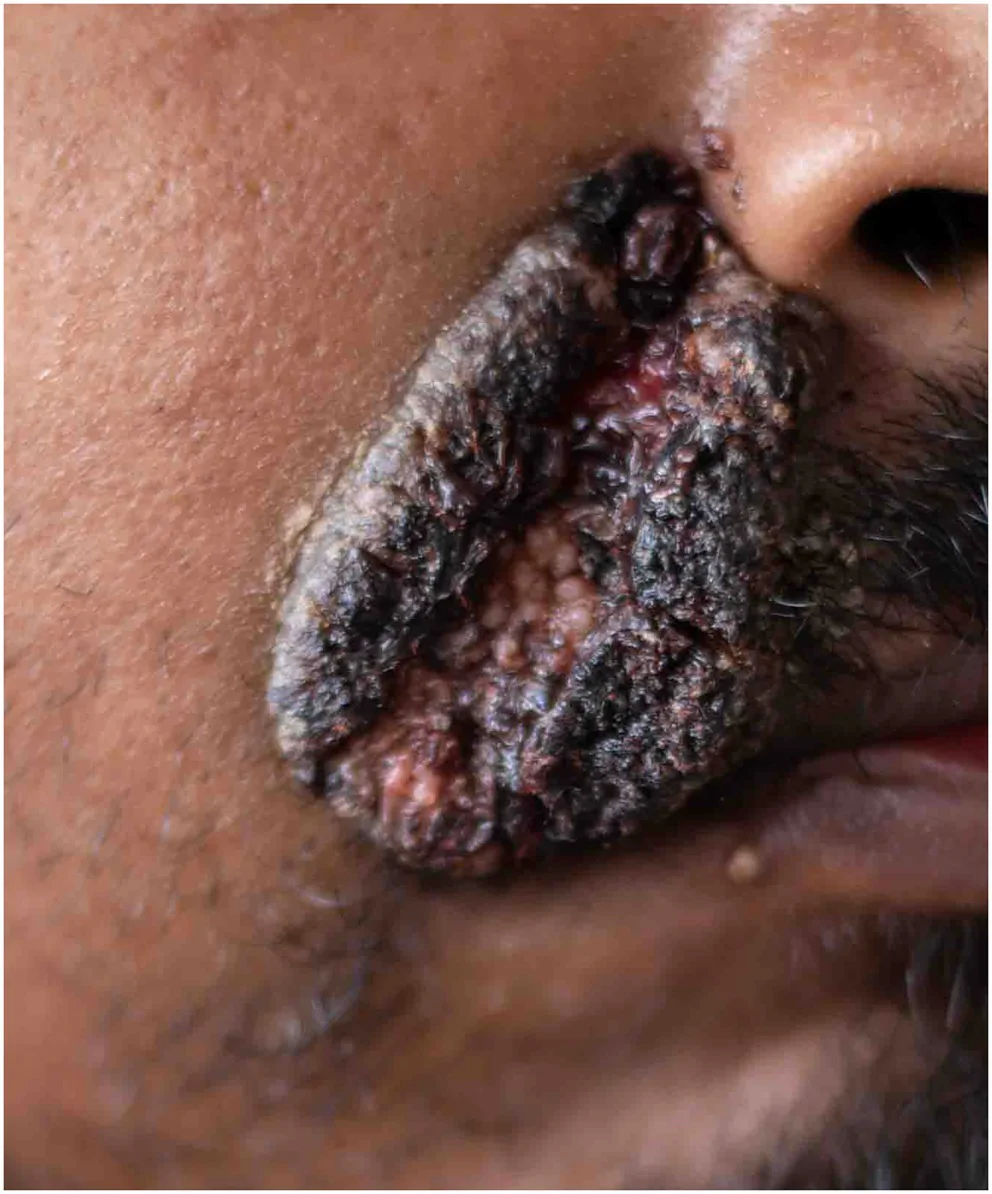
Clinical photograph of the facial lesion.

Maxillofacial computed tomography (CT) revealed an exophytic skin lesion arising from the right cheek and extending to the right nasolabial fold, with mild surrounding subcutaneous fat stranding and prominent reactive cervical lymph nodes. There was no evidence of osseous destruction or deep cervical involvement. Chest CT revealed numerous lung micronodules and an area of nodular consolidation in the left upper lobe, suggestive of possible pulmonary involvement in an underlying infectious etiology. The spleen demonstrated a heterogeneous appearance, raising concern for splenic involvement in disseminated infection. Laboratory evaluation demonstrated a positive serum *Blastomyces* antigen, and tissue culture from the facial lesion grew *Blastomyces dermatitidis*. Histopathologic examination of the biopsy specimen from the facial lesion revealed granulomatous lesions with broad‐based budding yeast within the dermis, consistent with *Blastomyces* spores (Figure 2). Together, these microbiologic, histopathologic, and imaging findings established the patient's diagnosis of disseminated blastomycosis with pulmonary, cutaneous, and splenic involvement.

**FIGURE 2:**
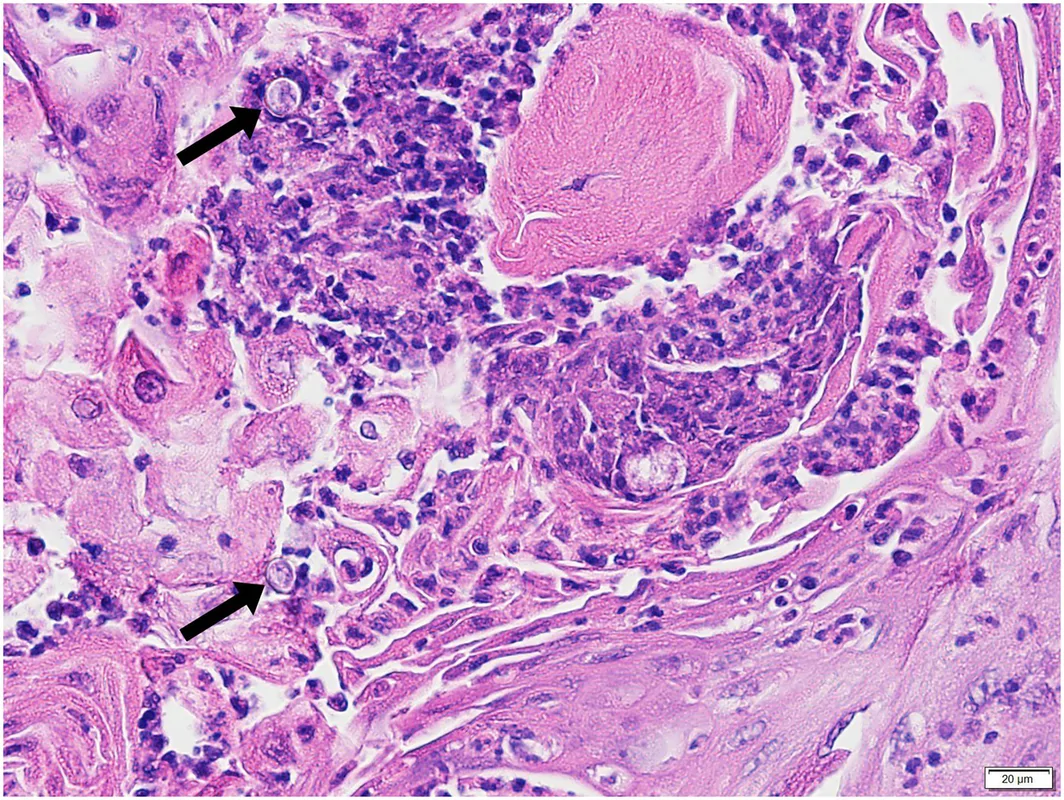
Histopathologic examination of the right facial lesion (hematoxylin and eosin stain), demonstrating granulomatous lesions with *Blastomyces* spores within the dermis, which are indicated by black arrows.

The patient was initiated on liposomal amphotericin B induction therapy (5 mg/kg intravenous daily for 6 days). Broader infectious work‐up and immunodeficiency screening were negative. The facial lesion improved during hospitalization, and the patient was discharged home with a transition to oral itraconazole (200 mg three times daily for 72 h, then twice daily). On outpatient follow‐up, he remained clinically stable. Repeat chest CT showed stable to improving lung nodules. Follow‐up serum *Blastomyces* antigen was negative.

## 3 Discussion

Blastomycosis is typically acquired by inhalation of aerosolized conidia in mold form following soil disruption [1]. Once inside the host's lungs, the fungus morphologically transitions into pathogenic yeast [1]. Previous studies have demonstrated that blastomycosis can significantly infect both immunocompromised and immunocompetent hosts, as seen in our patient [2, 5]. The host's immune status does not affect the extent of dissemination, likely reflecting the intrinsic ability of *Blastomyces* to evade host immunity. Because the primary route of entry for *Blastomyces* is through the lungs, blastomycosis is commonly misdiagnosed as community acquired pneumonia; however, blastomycosis is a condition for which antibiotic therapy is ineffective [6]. Extrapulmonary organ involvement through hematogenous spread occurs in 20%–50% of cases, with the skin being the most frequent site of dissemination, followed by the bones and genitourinary system [7, 8, 9]. Splenic abscesses and splenomegaly secondary to blastomycosis have also been rarely reported in the literature [10]. In our patient, the heterogeneous appearance of the spleen on imaging in the setting of disseminated blastomycosis raised concern for splenic dissemination, potentially representing an uncommon site of hematogenous dissemination [10]. The splenic changes observed in our patient may have also contributed to the decreased appetite and weight loss that he had been experiencing at the time.

Cutaneous blastomycosis most often arises secondary to pulmonary dissemination, though direct inoculation of the fungus through disrupted skin can also occur [11]. The lesions are commonly verrucous or ulcerative plaques with irregular borders. However, the clinical presentation is variable and can be mistaken for malignancy, nonhealing wounds, bacterial abscesses, or other infectious causes [12, 13, 14]. This variability often contributes to diagnostic delays and misdirected antibiotic therapy. However, heightened clinical suspicion and awareness of occupational and environmental risk factors can enable clinicians to recognize cutaneous manifestations of systemic blastomycosis.

Prior reports of disseminated blastomycosis with cutaneous involvement have highlighted the potential for delayed diagnosis because of its nonspecific clinical appearance. Strain et al. [15] described pulmonary blastomycosis with secondary cutaneous involvement in a 57‐year‐old female whose diagnosis was delayed approximately one year, emphasizing the diagnostic value of recognizing the cutaneous manifestation of this disease. More recent studies have likewise described cutaneous blastomycosis initially attributed to certain bacterial, inflammatory, and malignant skin conditions, such as bacterial folliculitis, pyoderma gangrenosum, and nonmelanoma skin cancer, resulting in delayed antifungal treatment [16]. Our case parallels these reports, as multiple courses of antibiotics were initially administered for our patient's facial lesion, delaying accurate diagnosis and treatment of disseminated disease. Given that this delay is common, a high index of suspicion is essential for accurate diagnosis.

Disseminated blastomycosis should be suspected in individuals who live in regions endemic for *Blastomyces dermatitidis* or *Blastomyces gilchristii*, including areas around the Ohio and Mississippi River valleys and Great Lakes, and who work in soil‐disrupting occupations, particularly those presenting with pulmonary and cutaneous disease [1]. Our patient had recently relocated from Mexico to Wisconsin. Although the precise location of acquisition cannot be definitely established, Wisconsin is a well‐recognized endemic region for blastomycosis, and the patient's construction work involving frequent soil and debris exposure provides a plausible local source of infection [1]. Therefore, the epidemiologic history favors acquisition in Wisconsin, though acquisition prior to relocation cannot be completely excluded. Notably, one study reported that the largest documented outbreak of blastomycosis in the US, including 131 cases at a paper mill, was attributed to both work and environmental exposure [17]. Detailed occupational history can guide accurate diagnosis of pulmonary and disseminated blastomycosis.

Definitive diagnostic test of blastomycosis and disseminated disease is fungal culture of the clinical specimen, including sputum, bronchoalveolar lavage, or tissue biopsies [1]. The histologic characteristic of blastomycosis is broad‐based budding yeast, which can be visualized with direct microscopy [1]. As seen in our patient's biopsy specimen, mixed neutrophilic and granulomatous inflammation often characterizes the immune response that accompanies the broad‐based budding yeast. Other diagnostic modalities can be utilized to guide diagnosis as well. Detection of serum or urine *Blastomyces* antigen has high sensitivity, but cross‐reactivity can occur [18, 19, 20]. Notably, while the sensitivity of urine antigen testing has been reported as high as 92.9%, the specificity is lower because of significant cross‐reactivity with other endemic mycoses such as histoplasmosis [18, 20].

For mild to moderate disease, the recommended treatment includes oral itraconazole, as outlined by the Infectious Diseases Society of America [21]. For moderately severe to severe disease or dissemination to the central nervous system (CNS), treatment includes liposomal amphotericin B therapy until clinical improvement, followed by oral itraconazole for a treatment duration of at least 12 months, compared with 6–12 months for mild to moderate disease [21]. Individuals with disseminated blastomycosis should undergo antifungal therapy, regardless of surgical resection, as studies have shown that itraconazole and amphotericin B achieve clinical cure and prevent relapse in disseminated disease [20, 21].

## 4 Conclusion

This case underscores the significance of obtaining a thorough occupational and environmental history when evaluating patients for possible infectious processes. Due to their frequent exposure to soil and decaying matter, construction workers are at risk for blastomycosis, which may present with dissemination and atypical organ involvement, particularly the spleen. Cutaneous lesions can also be valuable clinical clues to underlying systemic fungal infections. High clinical suspicion, accurate diagnosis, and prompt initiation of antifungal treatment are essential to prevent life‐threatening complications of disseminated blastomycosis.

## Author Contributions

**Toan N. Vu:** writing – original draft, writing – review and editing, conceptualization, visualization, methodology, formal analysis. **Nilay Kumar:** writing – review and editing, supervision, resources, validation.

## Funding

The authors have nothing to report.

## Ethics Statement

The authors have nothing to report.

## Consent

Written informed consent was obtained from the patient for publication of this case report and any accompanying images. A copy of the consent is available on record.

## Conflicts of Interest

The authors declare no conflicts of interest.

## Data Availability

Data sharing not applicable to this article as no datasets were generated or analyzed during the current study.
